#  Consolidated Health Economic Evaluation Reporting Standards 2022 (CHEERS 2022) statement: updated reporting guidance for health economic evaluations

**DOI:** 10.1136/bmj-2021-067975

**Published:** 2022-01-11

**Authors:** Don Husereau, Michael Drummond, Federico Augustovski, Esther de Bekker-Grob, Andrew H Briggs, Chris Carswell, Lisa Caulley, Nathorn Chaiyakunapruk, Dan Greenberg, Elizabeth Loder, Josephine Mauskopf, C Daniel Mullins, Stavros Petrou, Raoh-Fang Pwu, Sophie Staniszewska

**Affiliations:** 1 School of Epidemiology and Public Health, University of Ottawa, Ontario, Canada; Institute of Health Economics, Alberta, Canada; 2 Centre for Health Economics, University of York, York, UK; 3 Health Technology Assessment and Health Economics Department of the Institute for Clinical Effectiveness and Health Policy (IECS-CONICET), Buenos Aires; University of Buenos Aires, Buenos Aires; CONICET (National Scientific and Technical Research Council), Buenos Aires, Argentina; 4 Erasmus School of Health Policy & Management, Erasmus University Rotterdam, Rotterdam, The Netherlands; 5 London School of Hygiene and Tropical Medicine, London, England, UK; 6 Adis Journals, Springer Nature, Auckland, New Zealand; 7 Department of Otolaryngology - Head & Neck Surgery, University of Ottawa, Ontario, Canada; Clinical Epidemiology Program and Center for Journalology, Ottawa Hospital Research Institute, Ontario, Canada; Department of Epidemiology, Erasmus University Medical Center Rotterdam, Rotterdam, The Netherlands; 8 Department of Pharmacotherapy, College of Pharmacy, University of Utah, Salt Lake City, Utah, USA; 9 Department of Health Policy and Management, School of Public Health, Faculty of Health Sciences, Ben-Gurion University of the Negev, Be’er-Sheva, Israel; 10 Harvard Medical School, Boston, MA, USA; *The BMJ*, London, UK; 11 RTI Health Solutions, RTI International, Research Triangle Park, NC, USA; 12 School of Pharmacy, University of Maryland Baltimore, Baltimore, MD, USA; 13 Nuffield Department of Primary Care Health Sciences, University of Oxford, Oxford, UK; 14 National Hepatitis C Program Office, Ministry of Health and Welfare, Taipei City, Taiwan; 15 Warwick Research in Nursing, University of Warwick Warwick Medical School, Warwick, UK

## Abstract

Health economic evaluations are comparative analyses of alternative courses of action in terms of their costs and consequences. The Consolidated Health Economic Evaluation Reporting Standards (CHEERS) statement, published in 2013, was created to ensure health economic evaluations are identifiable, interpretable, and useful for decision making. It was intended as guidance to help authors report accurately which health interventions were being compared and in what context, how the evaluation was undertaken, what the findings were, and other details that may aid readers and reviewers in interpretation and use of the study. The new CHEERS 2022 statement replaces previous CHEERS reporting guidance. It reflects the need for guidance that can be more easily applied to all types of health economic evaluation, new methods and developments in the field, as well as the increased role of stakeholder involvement including patients and the public. It is also broadly applicable to any form of intervention intended to improve the health of individuals or the population, whether simple or complex, and without regard to context (such as health care, public health, education, social care, etc). This summary article presents the new CHEERS 2022 28-item checklist and recommendations for each item. The CHEERS 2022 statement is primarily intended for researchers reporting economic evaluations for peer reviewed journals as well as the peer reviewers and editors assessing them for publication. However, we anticipate familiarity with reporting requirements will be useful for analysts when planning studies. It may also be useful for health technology assessment bodies seeking guidance on reporting, as there is an increasing emphasis on transparency in decision making.

Economic evaluations of health interventions are comparative analyses of alternative courses of action in terms of their costs and consequences. They can provide useful information to policy makers, payers, health professionals, patients, and the public about choices that affect health and the use of resources. Economic evaluations are a particular challenge for reporting because substantial information must be conveyed to allow scrutiny of study findings. Despite a growth in published economic evaluations^1^ ^2^ ^3^ and availability of reporting guidance,^4^ there is a considerable lack of standardisation and transparency in reporting.^5^ ^6^ There remains a need for reporting guidance to help authors, journal editors, and peer reviewers in their identification and interpretation.

The goal of the original Consolidated Health Economic Evaluation Reporting Standards (CHEERS) statement,^4^ was to recommend the minimum amount of information required for reporting of published health economic evaluations. The statement consisted of a 24-item checklist and Explanation and Elaboration Report.^4^ CHEERS was intended to help authors provide accurate information on which health interventions are being compared and in what context, how the evaluation was undertaken, what the findings are, and other details that may aid readers and reviewers in interpretation and use of the study. In doing so, it can also aid interested researchers in replicating research findings. Some checklist items (such as title, abstract) were also included to aid those researching economic evaluation literature. The CHEERS statement consolidated previous health economic evaluation reporting guidelines^7^ ^8^ ^9^ ^10^ ^11^ ^12^ ^13^ ^14^ ^15^ ^16^ ^17^ ^18^ into one current, useful reporting guidance.

Since the original publication of the CHEERS statement, there have been several developments that have motivated an update. These include feedback on perceived limitations of CHEERS, including criticism of its neglect of addressing reporting of cost-benefit analyses.^19^ CHEERS has also been observed to be used inappropriately, as a tool to assess quality of methods, for which other tools exist,^20^ rather than the quality of reporting.^5^ It has also been used as a tool to quantitatively score studies in systematic reviews, an approach that could mislead readers and reviewers^21^ as it has not been designed for this purpose.

There have also been methods developments in economic evaluation motivating an update. This includes an update of methods proposed by the Second Panel on Cost-Effectiveness in Health and Medicine (“Second Panel”), which contained new recommendations concerning the perspective of economic evaluations, the classification of costs and benefits in a structured table, and the inclusion of related and unrelated healthcare costs in added years of life.^22^ Health technology assessment bodies have also updated their guidance on conducting and appraising economic evaluations.^23^ ^24^

There have also been increasing calls for the use of health economic analysis plans^25^ and the use of open source models.^26^ ^27^ ^28^ ^29^ ^30^ The latter may be of particular importance as published economic evaluations are increasingly available in journals with broad data-sharing policies. Increased use of, and guidance for, economic evaluations to support policy decisions in immunisation programmes^31^ ^32^ and global health in lower and middle income countries^33^ have also motivated an update. There has also been an increase in the number of economic evaluations that attempt to capture consequences extending beyond health outcomes, such as equity and distributional effects.^34^ ^35^

Finally, the increased role of stakeholder involvement in health research and health technology assessment, including patients and the public, suggests the need for reporting guidance to recognise a broader audience.^36^ ^37^ ^38^ All of these developments suggest the scope of guidance for reporting economic evaluations should be expanded and updated.

The objective of this article is to provide a brief overview of the CHEERS 2022 statement, which consists of a 28-item checklist, and an Explanation and Elaboration report with accompanying user tools and guidance. More detailed guidance and illustrative examples on how to use the checklist can be found in the larger Explanation and Elaboration report.^39^

**Summary points**

- To ensure health economic evaluations are interpretable and useful for decision making, authors need to provide sufficient detail about the healthcare context and decision under investigation, analytic approach, and findings, and the potential impact on patients, service recipients, and public or application in policy or patient care.
- This article provides a brief overview of the CHEERS 2022 statement, which provides updated reporting guidance that reflects the need for a broader application to all types of health economic evaluation and health interventions, new methods and developments in the field, as well as the increased role of participation from patients, service recipients, and other key stakeholders.
- The CHEERS 2022 statement consists of a 28-item checklist, and an Explanation and Elaboration report with accompanying user tools and guidance.
- The CHEERS 2022 statement is intended to be used for any form of health economic evaluation and is primarily intended for researchers reporting economic evaluations for peer reviewed journals as well as the peer reviewers and editors assessing them for publication. The statement is not intended as a scoring tool or a tool to assess the appropriateness of methods.
- Budget impact analyses and constrained optimisation studies are beyond the scope of the guidance.
- We anticipate familiarity with reporting requirements will be useful for analysts when planning studies and useful for health technology assessment bodies seeking guidance on reporting, as there is an increasing emphasis on transparency in decision making.

## Approach

The process of revising CHEERS followed that of ISPOR Good Practices Task Force reports^40^ as well as guidance developed by the Enhancing the QUAlity and Transparency Of health Research (EQUATOR) network,^41^ where the CHEERS 2022 update is also registered. An informal review was undertaken of reporting guidelines published since CHEERS, and new items were proposed and consolidated along with the existing CHEERS Checklist. In parallel with this, a task force was convened and a group of patient and public involvement and engagement (PPIE) contributors was formed to review the consolidated checklist and provide suggestions on language and the need for additional items. The draft checklist was finalised by CHEERS Task Force members.

Experts in economic evaluation, as well as those with perspectives in journal editing, decision making, health technology assessment, and commercial life sciences were invited to participate in a modified Delphi Panel (“Delphi”) process. Further details on how the Task Force and PPIE members were chosen is available in the Explanation and Elaboration document.^39^ Panellists along with the PPIE contributors were subsequently invited to participate by email and directed to a web based survey. Feedback from each round of the Delphi process was discussed by Task Force members, who ultimately finalised the checklist based on the input provided. A guiding principle for CHEERS is that economic evaluations made available publicly should be understandable, interpretable, and replicable to those who use them.

A completed Guidance for Reporting Involvement of Patients and the Public-Version 2 (GRIPP2)^42^ checklist is in Appendix A. The protocol for the Delphi process, as well as panel composition, size, response rates, and analytic approach can be found in Appendix B.

## The CHEERS 2022 statement

### Scope

The CHEERS 2022 statement is intended to be used for any form of health economic evaluation.^43^ This includes analyses that only examine costs and cost offsets (that is, cost analysis) or those that examine both costs and consequences. The latter include analyses that consider health consequences (such as, cost-effectiveness/utility analyses (CEAs/CUAs), cost minimisation, cost-benefit/benefit-cost analyses (CBAs)), and broader measures of benefit and harm to individuals (such as extended CEAs/CBAs), including measures of equity (such as distributional CEAs). While we are aware some studies comparing costs are labelled as CBAs, we recommend the use of this term for studies which include a monetary valuation of health outcomes. Although linked to economic evaluation, budget impact analyses and constrained optimisation studies are beyond the scope of CHEERS guidance, as they require additional reporting that addresses population dynamics and feasibility constraints and are addressed in other guidance reports.^44^ ^45^

The primary audiences for the CHEERS 2022 statement are researchers reporting economic evaluations as well as peer reviewers and editors assessing them for publication. While the statement is not intended to guide the conduct of economic evaluation, familiarity with reporting requirements will be useful for analysts when planning studies. CHEERS may be similarly useful for health technology assessment bodies seeking guidance on reporting, as there is an increasing emphasis on transparency in decision making.^46^ Health technology assessment and the use of economic evaluation is also becoming more commonplace globally.^3^ In developing the guidelines, the CHEERS Task Force considered issues that may be specific to regions with developing economies and healthcare systems, including providing examples of these by item in the larger report,^39^ to ensure the reporting guidance will be useful in any social or political context.

CHEERS is relevant for any intervention intended to affect health and should also be widely applicable for both simple and complex interventions, including programmes of care involving researcher-driven or commercialised products (such as drugs, macromolecules, cell, gene, and tissue based therapies, vaccines, and medical devices); public health and social care interventions; processes of care (such as e-health, care coordination, clinical decision rules, clinical pathways, information and communication, medical and allied health services); and re-organisation of care (such as insurance redesign, alternative financing approaches, integrated care, scope of practice change, and workplace interventions).

CHEERS is also applicable to studies based on mathematical modelling or empirical research (such as patient level or cluster level human studies). Although CHEERS can be used for systematic reviews of economic evaluation, its use should be limited to assessing the quality of reporting of a study rather than the quality of its conduct. As there is no validated scoring system for the checklist, using it as a scoring tool could lead to misleading findings and is strongly discouraged.^21^ If used to assess the quality of reporting in a systematic review, a qualitative assessment of completeness of reporting by item is a more appropriate approach. When applying the CHEERS statement, users may need to refer to additional reporting guidance (for example, for randomised controlled trials, patient and public involvement, modelling, health state preference measures), and these are referenced throughout the Explanation and Elaboration report.^39^

### How to use CHEERS

The CHEERS 2022 statement (checklist and Explanation and Elaboration report) replaces the 2013 CHEERS statement, which should no longer be used. The new CHEERS checklist contains 28 items with accompanying descriptions (table 1). Major changes from CHEERS 2013 are described in box 1. Checklist items are subdivided into seven main categories: (1) Title; (2) Abstract; (3) Introduction; (4) Methods; (5) Results; (6) Discussion; and (7) Other relevant information. Users of the checklist should first consult the Explanation and Elaboration report^39^ to ensure the appropriate interpretation of each item description.

**Table 1:** The CHEERS 2022 checklist

Section/topic	Item No	Guidance for reporting	Reported in section
**Title**			
Title	1	Identify the study as an economic evaluation and specify the interventions being compared.	
**Abstract**			
Abstract	2	Provide a structured summary that highlights context, key methods, results, and alternative analyses.	
**Introduction**			
Background and objectives	3	Give the context for the study, the study question, and its practical relevance for decision making in policy or practice.	
**Methods**			
Health economic analysis plan	4	Indicate whether a health economic analysis plan was developed and where available.	
Study population	5	Describe characteristics of the study population (such as age range, demographics, socioeconomic, or clinical characteristics).	
Setting and location	6	Provide relevant contextual information that may influence findings.	
Comparators	7	Describe the interventions or strategies being compared and why chosen.	
Perspective	8	State the perspective(s) adopted by the study and why chosen.	
Time horizon	9	State the time horizon for the study and why appropriate.	
Discount rate	10	Report the discount rate(s) and reason chosen.	
Selection of outcomes	11	Describe what outcomes were used as the measure(s) of benefit(s) and harm(s).	
Measurement of outcomes	12	Describe how outcomes used to capture benefit(s) and harm(s) were measured.	
Valuation of outcomes	13	Describe the population and methods used to measure and value outcomes.	
Measurement and valuation of resources and costs	14	Describe how costs were valued.	
Currency, price date, and conversion	15	Report the dates of the estimated resource quantities and unit costs, plus the currency and year of conversion.	
Rationale and description of model	16	If modelling is used, describe in detail and why used. Report if the model is publicly available and where it can be accessed.	
Analytics and assumptions	17	Describe any methods for analysing or statistically transforming data, any extrapolation methods, and approaches for validating any model used.	
Characterising heterogeneity	18	Describe any methods used for estimating how the results of the study vary for subgroups.	
Characterising distributional effects	19	Describe how impacts are distributed across different individuals or adjustments made to reflect priority populations.	
Characterising uncertainty	20	Describe methods to characterise any sources of uncertainty in the analysis.	
Approach to engagement with patients and others affected by the study	21	Describe any approaches to engage patients or service recipients, the general public, communities, or stakeholders (such as clinicians or payers) in the design of the study.	
**Results**			
Study parameters	22	Report all analytic inputs (such as values, ranges, references) including uncertainty or distributional assumptions.	
Summary of main results	23	Report the mean values for the main categories of costs and outcomes of interest and summarise them in the most appropriate overall measure.	
Effect of uncertainty	24	Describe how uncertainty about analytic judgments, inputs, or projections affect findings. Report the effect of choice of discount rate and time horizon, if applicable.	
Effect of engagement with patients and others affected by the study	25	Report on any difference patient/service recipient, general public, community, or stakeholder involvement made to the approach or findings of the study	
**Discussion**			
Study findings, limitations, generalisability, and current knowledge	26	Report key findings, limitations, ethical or equity considerations not captured, and how these could affect patients, policy, or practice.	
**Other relevant information**			
Source of funding	27	Describe how the study was funded and any role of the funder in the identification, design, conduct, and reporting of the analysis	
Conflicts of interest	28	Report authors conflicts of interest according to journal or International Committee of Medical Journal Editors requirements.

Box 1 **Major changes in the CHEERS 2022 statement (compared with CHEERS 2013)**

- Items related to patients or service recipients, the general public, and community or stakeholder involvement and engagement added.
- Language broadened to make CHEERS more widely applicable to cost-benefit/benefit-cost analysis, as well as equity or distributional cost effectiveness.
- Item related to reporting and availability of a health economic analysis plan added.
- Item related to characterising distributional effects added.
- Items distinguishing between model based and study based measures removed.
- Recommendation to report where publicly available models can be found added. Sharing of unlocked models with editors and reviewers encouraged.

Those using the checklist should indicate the section of the manuscript where relevant information can be found. If an item does not apply to a particular economic evaluation (for example, items 11-13 for cost analyses, or items 16 and 22 for non-modelling studies), checklist users are encouraged to report “Not applicable.” If information is otherwise not reported, checklist users are encouraged to write “Not reported.” Users should avoid the term “Not conducted” as CHEERS is intended to guide and capture reporting.

As before, in developing the CHEERS Statement, the Task Force recognises that the amount of information required for adequate reporting will exceed conventional space limits of most journal reports. Therefore, in making our recommendations, we assume that authors and journals will make necessary information available to readers using online and supplementary appendices or other means.

In addition to the open access Explanation and Elaboration report,^39^ we have also made available templates, an interactive form (https://don-husereau.shinyapps.io/CHEERS/), and further educational materials for authors, to facilitate appropriate use of the guidance. We encourage authors to visit the CHEERS^47^ and EQUATOR^48^ websites to locate copies of the checklist, the Explanation and Elaboration report,^39^ links to educational resources, templates, translations, a link to the interactive form and future updates.

## Discussion

We hope this update of the CHEERS statement will be useful to those who need to identify, prepare and interpret reports of health economic evaluations. Despite the promotion and increased number of available health economic evaluations, as well as the availability of CHEERS in multiple languages since 2013, there is some indication CHEERS could be more widely and appropriately used. A convenience sample of 50 articles citing CHEERS revealed only 42% (95% confidence interval 28% to 56%) made an appropriate use of CHEERS.^5^ This is a similar rate to those observed with other major reporting guidelines (CONSORT, PRISMA, ARRIVE). The same study also found that the inappropriate use of CHEERS has increased from its time of publication.

In creating this update, we also wanted to ensure the broadest possible application of CHEERS. Previous concerns raised about its lack of applicability in cost-benefit analyses (CBAs) were understandable, given original CHEERS guidance leaning strongly towards proving direction for those conducting cost-effectiveness analyses (including cost-utility analyses). This was driven, in part, by the small prevalence and impact of published CBAs at the time of the original CHEERS guidance. However, it is clear that broader characterisations of the benefits of healthcare, in concert with the promotion and publication of other forms of economic evaluation, such as distributional cost-effectiveness analysis, are becoming increasingly important. Health economic evaluation is also finding increasing application across a wider spectrum of health interventions. We hope the revised CHEERS statement addresses these concerns.

We are also aware that the final checklist reflects the perspectives of the Task Force members, PPIE advisors, Delphi Panel members, and peer reviewers involved. While nominal group techniques such as the Delphi approach are intended to minimise the excessive influence of dominant experts in a group, we acknowledge the output of these processes are only as good as the experience and perspectives represented. While a diversity of expertise was sought, it is possible that more could be said for specific applications of CHEERS for interventions that have impacts beyond health (for example, educational, environmental, social care). We would encourage those who see opportunities to expand CHEERS 2022 items, or to create additional reporting guidance that provides clarification in specific areas, to work with members of the CHEERS Task Force to develop CHEERS extensions in these areas.

The updated guidance also anticipates future developments in the conduct and reporting of published health economic evaluations. These include the use of health economic analysis plans, model sharing, and the increasing involvement of stakeholders in health research, including engagement with communities, patients, and the public. While some on the Delphi Panel suggested that these developments did not warrant their own reporting items, the Task Force ultimately felt addressing these developments through the creation of separate items could foster awareness of their use and development.

As there is an increasing need for clarity of information to support healthcare decision making and attention to healthcare expenditure, we anticipate the role of published health economic evaluation to become more important. While we hope the CHEERS 2022 statement and accompanying resources will ultimately improve the quality of reporting (and decision making), the impact of the original CHEERS statement on reporting quality is still uncertain. A formal evaluation study is ongoing, and results will be available in 2022.^49^ In the meantime, we have focused our attention on strategies to increase the appropriate use of CHEERS, including creating a wider range of tools and resources for editors and authors, seeking endorsement across a larger group of journals, and increasing outreach efforts.

We also recognise that researchers may wish to translate CHEERS 2022 into other languages. In these cases, we would encourage appropriate methods^41^ ^50^ and collaboration with Task Force members to ensure consistency with CHEERS. We encourage authors, peer reviewers, and editors to regularly consult the CHEERS 2022 webpage and to provide feedback on how it can be improved.

### Conclusion

This summary article presents the new CHEERS 2022 28-item checklist, and recommendations for each item. The CHEERS 2022 statement is primarily intended for researchers reporting economic evaluations for peerreviewed journals as well as the peer reviewers and editors assessing them for publication. However, we anticipate familiarity with reporting requirements will be useful for analysts when planning studies. It may also be useful for health technology assessment bodies seeking guidance on reporting, as there is an increasing emphasis on transparency in decision-making.

## Notes

Contributors: DH is Task Force co-chair and the submitting and corresponding author; MD is a Task Force co-chair. All other authors (FA, EBG, AHB, CC, LC, NC, DG, EL, JM, CDM, SP, RFP, and SS- listed in alphabetical order by surname) and the co-chairs conceived this paper and designed the Delphi survey conducted to inform the guideline content. DH conducted a literature review, administered the Delphi survey and analysed the data for both. DH and MD prepared materials for each meeting and led the drafting and editing of the article. DH, MD, FA, AHB, EBG, CC, LC, NC, DG, EL, JM, CDM, SP, RFP, and SS drafted particular sections of the article. MD and SS led the PPIE advisory group. DH and CC led the Editors Advisory Group. All authors were involved in revising the article critically for important intellectual content. All authors approved the final version of the article. DH is the guarantor of this work. The corresponding author attests that all listed authors meet authorship criteria and that no others meeting the criteria have been omitted.

David Moher served as scientific advisor and reviewed the initial proposal as well as attended initial meetings; all members of the PPIE (Ivett Jakab, Emma Kinloch, Eric Low, Jean Mossman, Declan Noone, Phil Posner, and Jo Watson) critically reviewed the initial checklist and suggested and provided review and advice on subsequent drafts of the checklist and Explanation and Elaboration report. Members of the PPIE were invited to participate in the Delphi Panel exercise. All members of the PPIE, and Delphi Panel were invited to review drafts of the checklist and accompanying report.

Provenance and peer review: Not commissioned; externally peer reviewed.

## Web Extra

Extra material supplied by the author

Appendix A: A completed Guidance for Reporting Involvement of Patients and the Public-Version 2 (GRIPP2) checklist

Appendix B: Protocol for the Delphi process
