# Supplementary material for: Consolidated Health Economic Evaluation Reporting Standards 2022 (CHEERS 2022) statement: updated reporting guidance for health economic evaluations
Source: BMJ. 2022 Jan 11;376:e067975. doi: 10.1136/bmj-2021-067975 (PMC8749494; doi:10.1136/bmj-2021-067975)
Supplement: Supplementary file 1 — Appendix A: A completed Guidance for Reporting Involvement of Patients and the Public-Version 2 (GRIPP2) checklist [file husd067975.wa.pdf]

Append A GRIPP2 – Short Form: Public Involvement in CHEERS

Aims	1. To establish an international public reference group to guide public involvement in CHEERS.<br>2. To embed public involvement at each key stage of the CHEERS checklist update.
Methods	We were unable to identify specific reporting guidance to guide the reporting of patient and public involvement and engagement (PPIE) in health economic evaluation, although GRIPP2 provides generic guidance on high quality PPIE reporting (Staniszewska et al 2017). Recognising the potential for PPIE in health economics research more generally, we identified the need for CHEERS to include items that enable reporting of PPIE and community engagement in health economic evaluation.<br><br>We established a public reference group made up of individuals with an interest in the reporting of health economic evaluation, who have knowledge of research and HTA and have been involved in a range of studies. We purposively selected individuals who would represent a public view, rather than a specific area of patient experience, as we recognised that the discussion about CHEERS items would happen at a macro level, rather than focusing on specific areas of patient experience.<br><br>We used a series of meetings or 'knowledge spaces' to create opportunities for deliberative dialogue about CHEERS. These meetings included the research team presenting on the background and development of CHEERS. In the first meeting we reviewed the CHEERS items with public contributors commenting on item wording and meaning. Each item was considered separately. The research team then edited the items, drafted new PPIE items and circulated that to the Public Reference group. These items were then discussed at the second meeting, prior to the Delphi exercise, ensuring PPIE was built in early in the process. In meeting three the focus was on reviewing progress, developing ideas for resources to support patient and public in dissemination of CHEERS. The draft paper, the document supporting PPIE in involvement in health economic evaluation and final checklist was sent to the PPIE Reference group for comment and input which was acted on.
Results	Each item was reviewed by the Public Reference Group and discussed with some editing to clarify meaning form a public perspective.<br><br>The Public reference Group identified the need for additional items to capture any patient and public involvement in a health economic evaluation. After discussion with the Public Reference group and with wider collaborators and following a process of editing and refinement, two key items were included in the checklist. These items are Items 21 and 25.<br><br>In addition to discussions about the items, the Public Reference Group also discussed the need to create resources that support patients and public contributors to engage in discussions about health economics. In response to input from the public reference group we developed a guide to support patient and public involvement in health economic evaluation, which the public reference group commented on, resulting in further refinements. For examples, they suggested we include all items rather than a selection of indicative items. This document will be published separately The Group also identified a range of dissemination approaches that would support public awareness and potential use of CHEERS.
Discussion	Public involvement in a health economic evaluation is in its infancy but it has the potential to contribute new forms of knowledge, important insights and ultimately enhance the validity, trustworthiness, legitimacy and accountability of health economic evaluation. The CHEERS PPIE items will enable the reporting of patient and public involvement and contribute to a strengthening evidence base underpinning health economic evaluation.

Reflections	CHEERS PPIE items will encourage reporting of involvement and engagement when it is carried out but also encourage health economics to consider the potential benefits of involving patients and the public in their work.
