# Supplementary material for: Consolidated Health Economic Evaluation Reporting Standards 2022 (CHEERS 2022) statement: updated reporting guidance for health economic evaluations
Source: BMJ. 2022 Jan 11;376:e067975. doi: 10.1136/bmj-2021-067975 (PMC8749494; doi:10.1136/bmj-2021-067975)
Supplement: Supplementary file 2 — Appendix B: Protocol for the Delphi process [file husd067975.wb.pdf]

Appendix B Protocol for Delphi Process

Objective

The overarching objective is to produce a minimum number of required reporting elements for health economic evaluations. It should be remembered that CHEERS is about the reporting of economic evaluations, that have been defined as ‘the comparative analysis of alternative courses of action in terms of both their costs and consequences.’ This will be done through capturing and conveying judgment from experts in health economic evaluation and medical journal editing.

Context and Rationale

Reports of economic evaluations in health care should be clear about why a study was required, how it was conducted (including its data sources and key assumptions), and what its findings and conclusions are. This is critical for it to be understood and useful to policymakers, providers, and patients. Moreover, these stakeholders need to interpret study findings.

Reporting guidelines that consist of a minimum number of required reporting elements have been proposed as a practical method of helping authors, editors, and peer-reviewers produce reports that convey details of a study transparently and consistently.   Which reporting elements in any scientific article are minimally required, however, is a matter of subjective opinion. Consensus methods such as a Delphi process[1,2] are useful to capture the minimum number of elements of reporting using a variety of expertise while minimizing the unnecessary influence of dominant experts in a group.

DO NOT COPY

Methods and timelines

Participants

Participants are expected to have expertise in

1) the conduct, analysis and interpretation of health economic evaluation; or
2) biomedical journal editing and reviewing.

It may be additionally important to invite those who can correctly apply the findings of studies including care providers, patients, members of the public and policy makers.
A purposive sample will be selected by Task Force Members who will individually approach potential participants through email. Potential participants will then have their participation confirmed by an official letter of invitation sent by the ISPOR Staff Liaison / Delphi Administrator.

Process

A modified Delphi process [2,3] will be conducted by a delegated member of the ISPOR CHEERS II Task Force. Participants will be asked to vote on the relevance of candidate reporting elements on a checklist. Reporting elements will be numbered and arranged under conventional ordering of sections for an economic evaluation report (e.g., title, abstract, introduction, methods, etc.).

Each item will have a reporting item description, an associated definition of what is meant by the item and a rationale for inclusion. An example is as follows:

Item: DISCOUNT RATE - Report the choice of discount rate(s) used for costs and outcomes and say why appropriate.
Definition: “Discount rate – an annual rate, described in percentage, used to calculate current values of resources or health outcomes”.

Rationale for inclusion: Rationale for inclusion: the discount rate reflects societal preferences for immediate gains in health or wealth versus gains that occur further into the future. The choice of discount rate affects the "present" value of costs and consequences that occur in the future. It is therefore important that the reader is aware of the discount rate chosen and the reasons for the choice, especially for programmes where the costs and consequences occur over several years.

An email reminder will be sent to participants 7 days and 4 days before the deadline for each round of the survey.

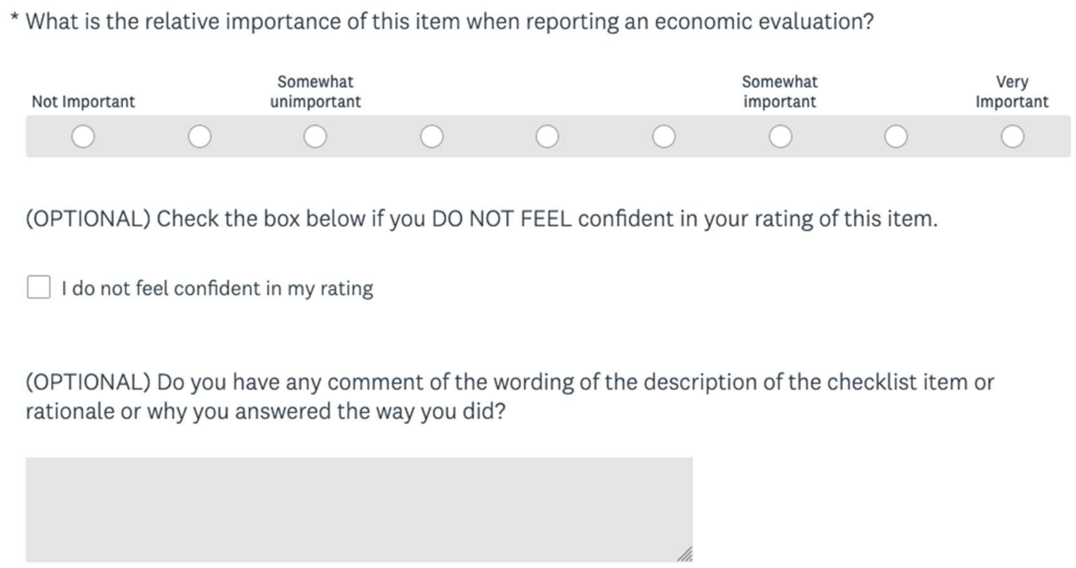
* What is the relative importance of this item when reporting an economic evaluation?

Not Important | Somewhat unimportant | Somewhat important | Very Important

(OPTIONAL) Check the box below if you DO NOT FEEL confident in your rating of this item.

I do not feel confident in my rating

(OPTIONAL) Do you have any comment of the wording of the description of the checklist item or rationale or why you answered the way you did?

Demographic information including, sex, age, location of work, region of work, and years of experience will also be collected.

Each participant's responses will be collated, and responses entered onto an Excel spreadsheet. Measures of central tendency and variability (mean and medians, with 95% CI and IQR distributions as well as a maximum-minimum range for each response, weighted by participant confidence) will be calculated. Participants will also have the opportunity to comment on the checklist items or to suggest additional items.

We a priori define a consensus-equivalent threshold of ≥70%, to support inclusion of an item into the second round. An item will be removed if more than 70% of the members score it as < 7 on the scale. Participants will be asked to comment on the wording and response options, and propose new items, if needed. A summary of the rating and feedback on each remaining item will be created.

In the second round, checklist items will be listed in order of importance, using the mean scores and median score, the inter-percentile range (IPR) (30th and 70th), and the inter-percentile range adjusted for symmetry (IPRAS), for each item ($_i$) being rated. (Fitch K, Bernstein S, et al.) from round one. Respondents will be asked to revisit their answer in light of these scores and revise. They will be asked to provide a rationale for revising their original estimate including evidence that they think is relevant. Participants will be told that items with a mean score of ≥ 7 will be grouped as 'Included' checklist items. Items with a mean score of > 4 and < 7 will be grouped as 'Possible' checklist items and might not be included in the final checklist.

Items with a mean score of 1 to 4 will be grouped as a 'Rejected' checklist list item. Rejected items will not be included in the final checklist unless they receive much higher scores in round two. Newly introduced items will also be voted upon this round.

If 'Possible' items still exist, a third round may be initiated. Again, checklist items will be listed in order of importance, using the mean scores from round one. Respondents will be presented with reasons for high and low estimates of importance. 'Possible' items that score do not score higher than 4 will be removed from the checklist. 'Possibles' that score above 5 will be included. Participants will then be invited to comment on which arguments they found unconvincing and why.

If required, the Delphi survey will then proceed to a final round, where respondents are presented with scores along with counterarguments against reasons for high and low estimates of importance, particularly for those items scored as 'Possible'. Participants are asked to create a final revision of their ratings.

In each round, respondents will be given 7 days to provide responses. Reminders will be sent out 3 days and 1 day before the deadline. A week will be then taken to analyze responses and accumulate any late responses. A flowchart of participation including number approached, and subsequently unavailable or non-responsive will be kept. In all analyses, sensitivity will be conducted by respondent self-reported confidence in their response. In some cases, mean values may be taken from those who were most confident, if there is a wide variation in responses reported.

Appendix Table B1 Process

Deliverables / Actions	Description	Date	Output
Discuss/Finalize Protocol	Committee Members to discuss-suggest additions to protocol at teleconference and subsequent emails	Aug, 2020	Final Protocol for Delphi
Finalize the Panel	Each Committee Member Identifies Individuals with Expertise for Potential Interest	May-June, 2020	List of 30-40 potential experts sent to Delphi Administrator
Gauge interest in participation	TF Member sends email to see if member is interested	Aug, 2020	List of interested participants
Invitation to participate	Administrator sends out official invitation to participate	Aug, 2020	Contact established with Delphi administrator. Final list of participants
Discuss/Finalize Delphi Questionnaire	Draft questionnaire sent to CHEERS TF members for comments	Aug, 2020	Final Questionnaire
1st Round of Delphi Process	Respondents asked to rate the importance of each element and rate their ability to answer	Sept, 2020	Complete first round of Delphi process
2nd Round of Delphi Process	Respondents asked to revisit their answer in light of importance and revise it. They will be asked to	Sept, 2020	Complete second round of Delphi process

	provide a rationale for revising their original estimate		
Possible 3rd Round of Delphi Process	Respondents presented with median and IQR along with reasons for high and low estimates of importance. Participants asked to specify which arguments they found unconvincing and why	Oct, 2020	Complete 3rd Round
Possible 4th Round of Delphi Process	Respondents presented with median and IQR along with counterarguments against reasons for high and low estimates of importance. Participants asked to revise	Oct, 2020	Draft Prepared
Acknowledgment	Respondents will be thanked and asked if they wish to be acknowledged in the Task Force publication	Oct, 2020	

Invitation Letter for Survey Nominees

Subject: ISPOR Task Force on Health Economic Evaluation Reporting Guidelines - Interest in Participating in a Delphi Panel survey

[Name],
I am wondering if you would be interested in participating in a Delphi panel survey being conducted by the International Society for Pharmacoeconomics and Outcomes Research (ISPOR).

DO NOT COPY

We are interested in capturing the opinions of 30-40 internationally recognized experts from [biomedical journal editing, health economic evaluation, patient experts, decision makers, industry] to develop a minimum set of standard reporting requirements for health economic evaluations. The minimum set will constitute a ‘checklist’ of reporting requirements.

The Delphi survey will be administered by email and begin in the third week of September and could require 60 minutes of your time in the first round. It is anticipated the survey will be repeated once (and possibly twice) every two to three weeks after this time.

It is hoped that the checklist will be published next year and if you wish, you could be acknowledged as a survey participant. If you are interested in participating in the survey, please let me know by [DATE], and I will pass on your name to ISPOR, who will send you an official invitation shortly.

Cheers,

[Don and Mike]

References

1 Moher D, Schulz KF, Simera I, *et al.* Guidance for developers of health research reporting guidelines. *PLoS Med* 2010;**7**:e1000217. doi:10.1371/journal.pmed.1000217

2 Fitch K, editor. *The Rand/UCLA appropriateness method user's manual*. Santa Monica: : Rand 2001.

3 Hasson F, Keeney S, McKenna H. Research guidelines for the Delphi survey technique. *J Adv Nurs* 2000;**32**:1008–15.

DO NOT COPY
